# Effects of Green Tea Extracts on the Pharmacokinetics of Quetiapine in Rats

**DOI:** 10.1155/2015/615285

**Published:** 2015-01-29

**Authors:** Essam Ezzeldin, Yousif A. Asiri, Muzaffar Iqbal

**Affiliations:** ^1^Department of Pharmaceutical Chemistry, College of Pharmacy, King Saud University, Riyadh 11451, Saudi Arabia; ^2^Bioavailability Laboratory, College of Pharmacy, King Saud University, Riyadh 11451, Saudi Arabia; ^3^Department of Clinical Pharmacy, College of Pharmacy, King Saud University, Riyadh 11451, Saudi Arabia

## Abstract

Quetiapine is an atypical antipsychotic, used clinically in the treatment of schizophrenia, acute mania in bipolar disorders, and bipolar depression in adults. In this study, the effect of green tea extracts (GTE) on the pharmacokinetics of quetiapine (substrate of CYP3A4) was investigated in rats. Male Wistar albino rats received GTE (175 mg/kg) or saline (control) by oral gavage for 7 days before a single intragastric administration of 25 mg/kg quetiapine. Plasma concentrations of quetiapine were measured up to 12 h after its administration by a validated ultraperformance liquid chromatography-tandem mass spectroscopy. Pretreatment with GTE produced significant reductions in the maximum plasma concentration and area under the curve of quetiapine by 45% and 35%, respectively, compared to quetiapine alone. However, GTE did not produce significant change in elimination half-life and oral clearance of quetiapine. This study concluded that GTE may decrease the bioavailability of quetiapine when coadministered.

## 1. Introduction

Consumption of green tea (*Camellia sinensis*) as beverages and dietary supplements is a common practiced habit worldwide. It contains high amount of the potent antioxidants polyphenols and has been associated with significant beneficial effects on human health including cardiovascular disease, cancer, obesity, and infection [1–7]. The results of* in vitro* studies suggest that green tea extracts (GTE) and/or the catechins, which are believed to be the major polyphenols of green tea, may affect the disposition of drugs by modulating the activities of drug metabolizing enzymes like CYP3A and uptake or efflux transporters, for example, P-glycoprotein (P-gp) [8–13]. These* in vitro* studies results were also supported by* in vivo* experiments in which GTE and/or epigallocatechin gallate (EGCG) exhibit significant pharmacokinetic interaction with coadministered drugs [13–20].

Quetiapine (QUE), a dibenzothiazepine derivative, is an atypical antipsychotic, indicated for the treatment of schizophrenia, acute mania in bipolar disorders, and bipolar depression in adults. It has been found to be effective as a monotherapy and as an adjunctive to a mood stabilizer [21–23]. After oral administration, QUE is rapidly absorbed with peak plasma concentrations achieved ranging from 1 to 2 hours. The absolute bioavailability of QUE is unknown in human, although it was lower than 10% in rats and monkeys and 8 to 30% in dogs, respectively, consequent to an extensive first pass effect. Plasma protein binding is approximately 83% and it exhibits linear pharmacokinetics in the clinical dose range [24, 25]. QUE is extensively metabolized by the cytochrome P450 (CYP) isoenzyme 3A4; hence coadministration of CYP 3A enzyme inducer, for example, phenytoin and thioridazine, increases the oral clearance of QUE whereas coadministration of ketoconazole and cimetidine (CYP 3A enzyme inhibitors) reduces it [26–28].

Previous study reported that the bioavailability of clozapine (also an atypical antipsychotic) is reduced by pretreatment of GTE in rat [19]. Since QUE is also substrate of CYP 3A enzyme and GTE alter the plasma concentration of CYP3A substrates, this study was designed to study the effect of GTE on QUE pharmacokinetics in rats.

## 2. Materials and Methods

### 2.1. Chemicals and Instruments

QUE was received gratis from Ranbaxy Laboratories, India, and risperidone was purchased from Sigma-Aldrich, USA. Green tea leaves extracts (*Camellia sinensis*) consisting of 14% polyphenol (44 mg) were obtained from General Nutrition Corporation, USA. Ethanol, ethyl acetate, and methanol were of HPLC grade obtained from Winlab Laboratory whereas formic acid and sodium hydroxide were of analytical grade obtained from BDH Laboratory, England. The apparatus used for analysis of QUE sample consisted of ACQUITY UHPLC system coupled to triple-quadruple tandem mass spectrometer Micromass Quattro Micro (Waters Corp., Milford, MA, USA).

### 2.2. Animal Experiments

Twelve male Wistar albino rats weighting from 180 to 220 g were obtained from the Animal Care and Use Centre, College of Pharmacy, King Saud University, Riyadh, Saudi Arabia. In an open level study, rats were randomly divided into two groups (6 in each) and served as control and treatment group, respectively. Rats in treatment group received GTE (175 mg/kg, oral) [19] for seven days whereas control group received normal saline (10 mL/kg). The rats were fasted for at least 12 hours (overnight) before the day of experiment. Blood samples (approximately 0.6 mL) were collected from the retroorbital plexus into heparinized microfuge tubes at different time intervals (0, 0.25, 0.5, 1, 1.5, 2, 3, 4, 6, 8, and 12 h) after administration of QUE (25 mg/kg, oral) in the morning at 8 a.m. in both groups. Plasma samples were harvested by centrifuging the blood at 15,000 ×g for 10 min and stored frozen at −80 ± 10°C until analysis.

### 2.3. Determination of QUE Concentration by UPLC MS/MS

QUE was determined by the in-house validated UPLC-MS/MS method using risperidone as internal standard. The chromatographic separation was performed on Acquity BEH C_18_ column (50 × 2.1 mm, i.d., 1.7 *μ*m, Waters, USA) maintained at 40°C temperature. The mobile phase was composed of ethanol-water-formic acid (80 : 20 : 0.1, v/v/v) at a flow rate of 0.3 mL/min. Triple-quadruple tandem mass spectrometer equipped with electrospray ionization (ESI) interface was used for analytical detection. The detection was performed in ESI positive mode using multiple reaction monitoring (MRM) by the ion transitions of* m/z* 384.13 > 253.00 for QUE and* m/z* 411.18 > 191.07 for IS, having dwell time of 0.161 s. The Mass Lynx software (Version 4.1, SCN 714) was used to control the UHPLC-MS/MS system and data was collected and processed using Target Lynx program. Sample preparation was processed by liquid-liquid extraction method using ethyl acetate as extracting solvent. The dried extracts were reconstituted in 200 *μ*L of ethanol and transferred into HPLC vials subjected to the analysis.

### 2.4. Data Analysis

All values are expressed as the mean ± standard error. A noncompartmental pharmacokinetic analysis was carried out using WinNonlin software to calculate the *C*_max⁡_, AUC, *T*_1/2_, *λz*, CL, and MRT. Statistical differences of the means were assumed to be significant when *P* < 0.05 by Student's unpaired *t*-test.

## 3. Results and Discussion

**Figure 1:**
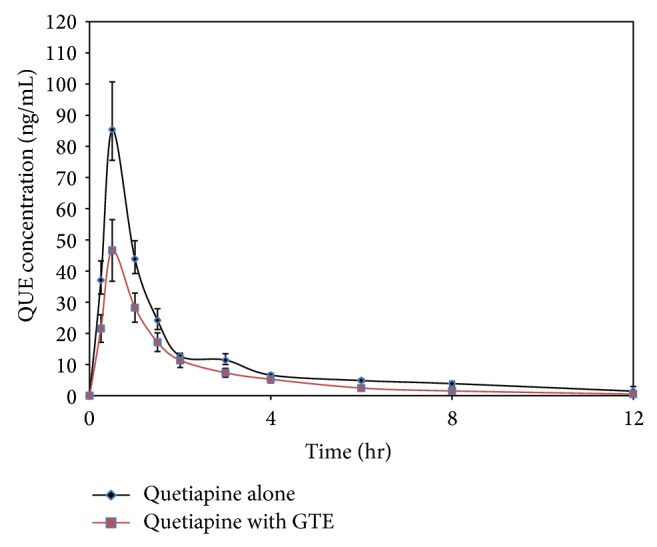
Plasma concentration-time curves of quetiapine (mean ± SE) after a single oral dose of 25 mg/kg with or without an oral dose of GTE 175 mg/kg.

Pharmacokinetic parameters results and plasma concentrations versus time profile of QUE with and without GTE administration are shown in Table 1 and Figure 1, respectively. Pretreatment with GTE (175 mg/kg) for seven days resulted in 45% decrease (*P* < 0.05) in *C*_max⁡_ of QUE without any change in *T*_max⁡_. Similarly, AUC_0–12_ and AUC_0–∞_ were also decreased (*P* < 0.05) by 35% and 38%, respectively, compared to control group. However, GTE did not produce any significant changes on *T*_max⁡_, elimination half-life, elimination rate constant, and clearance, suggesting that GTE did not affect the elimination of QUE and changes in bioavailability may be attributable to process that occurs in the gut rather than to a modification of systemic clearance. Previous studies showed that the coadministration of GTE and/or EGCG significantly increased the bioavailability of simvastatin, 5-FU, diltiazem, irinotecan, and tamoxifen [13, 14, 17, 18, 20], whereas it decreased the bioavailability of nadolol and clozapine [15, 19] in rats. In consequence to the above reports, our findings indicate that GTE decreases the bioavailability of QUE. GTE increased the bioavailability of tamoxifen, simvastatin, diltiazem, and irinotecan possibly by inhibition of CYP3A enzyme and/or P-gp. Although, like other atypical antipsychotics, QUE is not a substrate of P-gp [29], but it is major substrate of CYP3A4. In spite of that, GTE decreased the plasma concentration of QUE unexpectedly. In nadolol pharmacokinetic interaction study, it was concluded that the decrease in plasma concentration was produced possibly through unknown mechanism, which may include the inhibition of intestinal absorption mediated by uptake transporters, for example, OATP transporter not P-gp, whereas, in clozapine study, decrease in bioavailability was expected due to slight elevation in expression of hepatic CYP1A2m-RNA levels by GTE in rats. QUE is neither a CYP1A2 substrate nor P-gp and OATP transporter; in spite of that, its bioavailability was decreased. But it is also reported that the 7-hydroxy-N desalkyl quetiapine, a main active metabolite of QUE, was exclusively formed by CYP2D6 [30], and GTE also act as competitive inhibitor of human CYP2B6 and CYP2C8 in addition to CYP3A, especially in the intestine [9]. Hence, further study, in which quetiapine is needed to also be administered intravenously, might help to address this concern.

## 4. Conclusions

In conclusion, pretreatment with GTE significantly decreases the bioavailability of QUE (CYP3A4 substrate) in rats by unknown mechanism. No change in elimination rate and half-life raised the possibility of gut absorption site for GTE and QUE interaction. So, the mechanistic approach for the pharmacokinetic interaction between QUE and GTE needs to be investigated and care may be taken while taking green tea during QUE treatment, which may lead to therapeutic failure.

## Figures and Tables

**Table 1:** Pharmacokinetic parameters of QUE in rats after administration of a single oral dose of 25.0 mg/kg with and without GTE (175 mg/kg) (mean ± SE, *n* = 6).

Parameters	QUE alone (mean ± SE)	QUE with GTE (mean ± SE)
*C*_max⁡_ (ng/mL)	85.40 ± 34.22	47.49 ± 15.85^*^
*T*_max⁡_ (h)	0.5	0.5
AUC_0–24_ (ng·h/mL)	121.13 ± 32.59	78.16 ± 19.96^*^
AUC_0–*∞*_ (ng·h/mL)	137.10 ± 29.16	84.22 ± 18.91^*^
λz (h^−1^)	0.24 ± 0.11	0.34 ± 0.19
*T*_1/2_ (h)	3.49 ± 1.69	2.91 ± 2.22
CL (L/h/kg)	0.22 ± 0.04	0.34 ± 0.08
MRT (h)	3.50 ± 1.26	3.21 ± 1.69

^*^Significant difference from “QUE alone” group with *t*-test, ^*^ *P* ≤ 0.05; *C*_max⁡_: maximum plasma concentration; T_max⁡_: time to maximum plasma concentration; AUC: area under curve; *λz*: elimination rate constant; *T*_1/2_: elimination half-life; CL: clearance; MRT: mean residence time.
